# Impact of a Service-Learning Program Using Soccer Training on the Emotional and Behavioral Problems of Children with Developmental Disabilities

**DOI:** 10.3390/children11040467

**Published:** 2024-04-14

**Authors:** Huan Meng, Yonghwan Kim, Kyujin Lee

**Affiliations:** 1 Department of Physical Education, Gangneung-Wonju National University, Gangneung 25457, Republic of Korea; 20205047@gwnu.ac.kr (H.M.); yhkim@gwnu.ac.kr (Y.K.); 2 School of Social Integration, Adapted Physical Activity, Hankyong National University, Pyeongtaek 17738, Republic of Korea; 3 Institute of Sports Science, Seoul National University, Seoul 08826, Republic of Korea

**Keywords:** children with developmental disability, emotional and behavioral difficulties, service-learning program, exercise

## Abstract

Children with developmental disabilities may develop emotional and behavioral problems that arise from difficulties in social interaction, and therefore, a process of providing and testing intervention activities for emotional and behavioral issues in the early stages of development is required. This study evaluated the effects of a 12-week service-learning exercise program on children with developmental disabilities, contrasting it with a control group not engaged in specific physical activities. The participants included 18 children with developmental disabilities who participated in the service-learning exercise program (SLG) and 18 children with developmental disabilities (Non-SLG) who did not participate. The Korean Behavior Assessment System for Children-2 was used to evaluate children’s emotional and behavioral problems. A two-way repeated-measures ANOVA was conducted to verify the interaction effect between the group and period according to program participation. The results showed the positive internalization of problem behaviors in the emotional domain (F = 4.291, *p* = 0.011), attention deficit/hyperactivity (F = 4.761, *p* = 0.012), and personal adjustment (F = 4.116, *p* = 0.023) in the SLG group. These results suggest that participation in a service-learning exercise program positively affected emotional and behavioral problems in children with developmental disabilities. This could provide a basis for future research on service-learning exercise programs for children with developmental disabilities.

## 1. Introduction

Developmental disability refers to a disability that is characterized by the delayed or irregular development of intelligence, language, social skills, and motor skills [1]. As of June 2021, the number of individuals with developmental disabilities surveyed in Korea was 252,000, including 219,000 people with intellectual disabilities, an increase of approximately 7% compared to 2018 [2]. Children with developmental disabilities have difficulty engaging in the social interactions required to form relationships with others because they lack communication skills and frequently engage in stereotypical behavior, such as harming their own bodies or threatening or harassing others [3]. Because serious emotional and behavioral problems in children with developmental disabilities are likely to persist into adulthood, it is important to identify such issues early in their development and provide appropriate interventions [4].

Children with developmental disabilities typically have sensory and basic motor skill deficits that affect their motor development. To help develop physical skills in children with developmental disabilities, it is necessary to provide diverse opportunities for them to engage in physical activities appropriate for their developmental stage [5,6]. In terms of education, a range of physical activity interventions, such as physical activity, virtual reality intervention programs and aquatic exercises, have been tested and used for a long period as effective evidence-based interventions for children with developmental disabilities [7,8].

Given their multifaceted benefits, service-learning programs provide a unique opportunity to address the developmental needs of children with disabilities through structured physical activity and social engagement. Service learning applies the concepts of service and learning. It is defined as “a teaching and learning strategy that integrates meaningful community services through students’ education and reflective reflection” [9]. A service-learning program is not only an approach to integrate service and learning to strengthen each other’s benefits, but can also be said to be a multifunctional activity in that it meets the needs of the community and carries out educational activities to train experts within the university [10]. In addition, universities use service learning to instill students’ sense of social responsibility and achieve academic goals in the field of special education, which is being adopted to improve the educational environment for children with disabilities and to train experts [11]. In Korea, an experimental physical education class for children with disabilities was conducted using a service-learning program for children with developmental disabilities, and positive results were reported [12]. Although various service-learning programs for children with developmental disabilities are being implemented in the educational field, reports on the effects of exercise intervention for children participating in the programs are limited. In particular, research activities related to the emotional and behavioral problems of children with disabilities have become increasingly limited, and research activities for the emotional development of children with disabilities are required.

However, the reality is that children with developmental disabilities have fewer opportunities to participate in physical activities than non-disabled children, appropriate programs are rare, and it is more difficult for them to play team sports such as soccer. These problems are more complex than simply programming problems. Facilities and coaches and teachers with expertise in related fields must be prepared, and integrated programs with non-disabled children or a sufficient number of children with developmental disabilities must be recruited [13,14]. Service-learning programs have a multi-functional nature that can play a complementary role in addressing these issues.

Therefore, the purpose of this study was to compare and analyze the emotional and behavioral variables of a group of children with developmental disabilities who participated in a 12-week service-learning exercise program and a group that did not participate, and to determine the effect of participation in a service-learning exercise program on children with developmental disabilities. This study hypothesized that children with developmental disabilities who participate in a service-learning exercise intervention program would achieve greater improvement in their emotional and behavioral problems.

## 2. Materials and Methods

### 2.1. Research Procedures

To conduct this study, a researcher visited an educational institution operating a service-learning exercise program, consulted the institution’s officials, and provided them with a research guide and consent form. Participation in the study was limited to students whose parents provided consent, and inquiries about the research procedures and program were directed to the researcher’s contact information. This study evaluated the overall emotions and behaviors of research participants using the KBASC-2 to assess the impact of participation in a service-learning exercise program on the emotional and behavioral problems of children with developmental disabilities. The program participation group was limited to elementary school students with developmental disabilities who participated in the program at least once a week for 12 weeks. The non-participating group consisted of students who had not engaged in similar programs other than school physical education classes in the past six months. Although gender was not restricted, the children with developmental disabilities who ultimately participated in this study consisted of 38 boys. Participating children were evaluated 1:1 with a KBASC-2 expert pre-post program participation. The overall research progress was explained by the principal investigator. However, in order to exclude researcher bias, the same researcher classified and measured the groups, collected data, and anonymized the records.

This study protocol was approved by a university’s Institutional Review Board (IRB No. 1603/001-028) and informed consent for participation was obtained from all children and parents. There was no conflict of interest in this investigation.

### 2.2. Participants

Sample size was calculated by G*Power 3.1 (Universität, Düsseldorf, NRW, Germany): effect size, 0.25; α err prob, 0.05; power, 0.85; two group, and two tests. The results were a total sample size of 38 and an actual power of 0.85, as well as a Critical F of 4.11. This study was conducted on children with developmental disabilities (n = 38, age = 11.5 ± 2.4, only boys) in the 4th to 6th grades of elementary school using the convenience sampling method. This study employed the convenience sampling method due to logistical constraints, acknowledging that while this method facilitates participant recruitment in the specified region, it may limit the generalizability of the findings. They had not participated in sports programs other than physical education classes in the past six months. The inclusion criteria were (1) children diagnosed with an intellectual disability or autism by a doctor and (2) children who had received parental consent to participate in the study. The exclusion criteria were as follows: (1) having a medical condition or neurological disabilities beside developmental disabilities attributing to motor skill deficits; (2) participation in exercise regularly in the last three months; and (3) unreliable results from the questionnaire (i.e., using the same number to respond to all answers). This service-learning exercise program was based on a youth soccer education program. At the beginning of the study, 38 participants were included: 19 in the participating service-learning group (SLG) and 19 in the non-participating service-learning group (Non-SLG). However, one participant failed to continue with the 12-week service-learning exercise program regularly, and one failed to complete the pre- and post-tests. Therefore, the number of study participants used in the final data analysis was 36: 18 in the intervention group and 18 in the non-participating group (Table 1).

### 2.3. Emotional and Behavioral Assessment

This study used the Korean Behavior Assessment System for Children-2 (KBASC-2) to evaluate emotional and behavioral problems in elementary school students [15]. The KBASC-2 was selected for its comprehensive assessment of children’s emotional and behavioral problems, making it particularly suited for this study’s focus on developmental disabilities. Its multidimensional approach allows for a nuanced understanding of the impacts of the intervention The KBASC-2 is a multidimensional test tool that provides comprehensive information on children’s positive personality traits, social skills, adaptive resilience, and problem behavior patterns based on children’s self-perceptions and attitudes toward others. The KBASC-2 consists of 5 comprehensive scales and 20 subscales. The KBASC-2’s reliability and validity were verified by Ahn, Ebesutain, and Kamphaus [15], and it has been used as a tool for the evaluation of children’s emotions and behaviors in the South Korea elementary school environment. Cronbach alpha coefficients for the KBASC-2 were high, ranging from 0.85 (Inattention/Hyperactivity Composite) to 0.96 (Internalizing Problems Composite).

The KBASC-2 general criterion score is useful to summarize the entire test and to provide an extensive conclusion on the various types of personality traits and emotional and behavioral problems. Excluding the personal adaptation general criterion evaluating positive adaptation skills, a score of 60–69 is in the range of “high” on the sub-clinical level, and a score ≥ 70 is in the range of “very high” on the clinical level. In the personal adaptation general measure, a score of 31–40 is in the range of “low” on the subclinical level, and a score ≤ 30 is in the range of “very low” on the clinical level.

### 2.4. Soccer Training Program

The service-learning intervention program used in this study involved a physical activity program based on youth soccer skills. The service-learning exercise program consisted of physical coordination skills such as muscle coordination, balance, and reaction power, as well as basic skill training required for playing soccer. The program of this study consisted of four stages (Step 1, warm up; Step 2, body coordination; Step 3, technique; and Step 4, game]. In the first stage, the focus was to arouse interest in participating in children’s physical preparation and training to mitigate injury risks. In the second stage, training was conducted to foster balanced physical development through running, changing directions, and passing obstacles. In the third stage, skills such as dribbling, passing, and shooting were taught in an enjoyable manner. The fourth stage provided opportunities to achieve enjoyment through various game experiences, such as scoring and winning.

The participants attended a weekly class that included a service-learning exercise program leader (one person), an assistant leader (four people), and 20 students. The program level was reorganized according to the participants’ characteristics and level of motor function. Furthermore, the activity tasks were modified and presented through an individualized process for children with developmental disabilities. The program duration was 90 min per session and was conducted for 12 weeks, excluding pre- and post-evaluation. The specific activities carried out during the service-learning exercise program are summarized in Table 2.

### 2.5. Data Analysis

The mean and standard deviation were calculated for all data and were analyzed using IBM SPSS Statistics for Windows, version 28.0 (IBM Corp., Armonk, N.Y., USA). A normality test was performed to verify the homogeneity of the service-learning exercise program participants and non-participants, followed by an independent *t*-test. In addition, a two-way ANOVA for repeated measures was performed to verify the effect of the program participation and non-participation groups and the interaction effect between group and period. The statistical significance level for all results was set at less than 0.05.

## 3. Results

### 3.1. Emotional Variables of Children with a Developmental Disability

The analysis revealed that participation in the service-learning exercise program had a significant positive impact on several emotional and behavioral variables among children with developmental disabilities.

The changes in the comprehensive scale variables after 12 weeks of exercise were as follows: significant effects were found for internalizing problem behaviors (F = 4.291, *p* = 0.011), attention deficits, and problem behaviors (F = 4.761, *p* = 0.012). However, no significant difference was found in school-related problems (F = 0.287, *p* = 0.712). Looking at the detailed variables of school-related problems, there were no significant differences between attitudes toward school (F = 0.243, *p* = 0.765) and teachers (F = 0.381, *p* = 0.671). In the case of detailed internalization variables, locus (F = 7.214, *p* = 0.002), anxiety (F = 8.289, *p* < 0.001), depression (F = 7.191, *p* = 0.003), and sense of inactivity (F = 4.181, *p* = 0.018) showed significant differences; however, atypicality (F = 1.821, *p* = 0.311) and social stress (F = 3.911, *p* = 0.053) did not. For the detailed attention-deficit/hyperactivity variables, both inattention (F = 3.929, *p* = 0.031) and hyperactivity (F = 6.998, *p* = 0.007) showed significant differences. The changes in the emotional variables of children with developmental disabilities according to their participation in the service-learning exercise program are shown in Table 3.

### 3.2. Changes in Problem Behavior Variables of Children with a Developmental Disability

When examining the comprehensive scale variables after the 12-week exercise intervention, a significant effect was observed in terms of personal adjustment (F = 4.116, *p* = 0.023). Regarding the detailed variables of personal adjustment, we observed significant differences in interpersonal relations (F = 6.841, *p* = 0.004), self-esteem (F = 7.982, *p* = 0.002), and self-reliance (F = 6.921, *p* = 0.003). However, there was no significant difference in variables representing their relationship with their parents (F = 0.519, *p* = 0.691). Changes in children’s problem behavior variables according to their participation in the 12-week service-learning exercise program are summarized in Table 4.

## 4. Discussion

This study aimed to compare and analyze the emotional and behavioral problem variables of children with developmental disabilities who participated in a service-learning exercise program to determine the impact of participation in a service-learning exercise program on the emotional and behavioral problems of children with developmental disabilities. Based on the research results, the implications regarding the impact of service-learning exercise programs on the emotional and behavioral problems of children with developmental disabilities are discussed as follows.

No statistically significant changes were found in the variables related to school problems according to the participation in the service-learning exercise program. It is assumed that the lack of change in school problems, despite the positive change in the internalized problem behavior of children with disabilities, was due to two main reasons. First, no significant problems were found in the variables related to school problems among children with developmental disabilities before participating in the program, and weekly classes may have been insufficient to change the students’ attitudes. Similar results were obtained in a study by Lee et al. [16] that compared the emotional and behavioral problems of children with developmental coordination disorders and children without disabilities. Second, due to the nature of the after-school program outside school, the targets of activities, such as teachers and friends, changed. Consequently, it did not have an effect on attitudes related to school issues. In particular, in the case of children with developmental disabilities, attitudes toward school issues may differ depending on whether the children participate in special schools or special classes in general schools; therefore, it is necessary to control for additional variables at the school level in follow-up studies.

Children with developmental disabilities have negative experiences, such as being excluded from play or sports activities, which can be emotionally damaging for elementary school children, as they are an important part of childhood development [17]. Emotional variables such as a child’s locus of control, social stress, anxiety, and depression affect attitudes toward school and teachers, which can also cause problems at school [18]. These childhood problems persist into adolescence and adulthood; therefore, it is important to identify them early and provide appropriate intervention activities [19]. Various activities, such as occupational therapy, speech therapy, and art therapy, have been proposed as intervention activities for children with developmental disabilities; however, exercise intervention is effective in terms of accessibility to participation at school, at home, or in the community [20]. In particular, motor development has been reported as an intervention method for elementary school students who need to develop fundamental motor and specialized movement skills [8].

Analysis of the emotional variables of children with developmental disabilities following participation in a 12-week service-learning exercise program revealed positive changes in internalizing problem behaviors and attention deficit hyperactivity. This is in line with previous studies that reported a positive effect of exercise programs on internalizing problems such as anxiety, depression, attention deficit, and hyperactivity in children with disabilities [21,22]. To provide effective exercise to children with developmental disabilities, creating motivation and positive attention to physical activity is important [7]. The service-learning exercise program in this study continuously motivated students by organizing personalized classes that reflected the characteristics of children with developmental disabilities. One leader and four assistant instructors provided active feedback during the class to help students focus on the class. The organization of these programs and the provision of a class environment would have had a positive impact on the emotional variables of children with developmental disabilities.

Some children with developmental disabilities have communication limitations and behavioral problems arising from difficulties in social interaction [23]. Various exercise activities have been proposed to reduce stereotypical behavior, such as repeating the same movements or harming others [24]. In this study, there was a change in problem behavior of children with developmental disabilities after participating in service-learning activities. As a result of analyzing behavioral problem variables in children with developmental disabilities following participation in a 12-week service-learning exercise program, positive changes were found in interpersonal relations, self-esteem, and self-reliance on a personal adjustment scale. This supports the results of previous studies that reported positive changes in children with developmental disabilities through various exercise activities [23]. Sports activities effectively improve self-efficacy of people with disabilities in moving their body and achieving their goals [25]. In this context, it is believed that the exercise program used in this study positively affected the variables related to problem behaviors.

Exercise intervention plans for people with developmental disabilities must be developed while taking into account the characteristics of the developmental disabilities. In the meantime, in Korean educational settings, stretching, complex exercises, yoga, and water-related psychological exercises were implemented to help people with developmental disabilities improve their social adaptation and emotional stability [26,27,28]. The service-learning exercise intervention program implemented in this study diplsayed a positive effect compared to the results of previous studies, even though it was conducted relatively less frequently (once a week) compared to other exercise programs. This may be due to the service-learning program’s prior educational activities by leaders, the provision of an educational environment, and individualized programs to encourage physical activity in children with developmental disabilities [29].

Among the behavioral variables, there was no statistically significant change in the children’s relationships with their parents after participating in the exercise program. This may be because the service-learning exercise program did not include a program promoting bonding with parents. To address this problem, physical activity programs involving parents have been proposed [30]. Thomson et al. [23] reported improved parental awareness of physical activity through a participation program for children with developmental disabilities. An et al. [31] found that fostering cooperative relationships between teachers and parents positively impacted physical activity. Reflecting this point, follow-up research should consider ways in which to involve parents in programs to improve problem behaviors in children with developmental disabilities.

Despite the several novel insights uncovered here, this study may have some limitations. Therefore, caution is required when interpreting the results. First, because convenience sampling was used to recruit participants, the representativeness of the research group may be questioned. Second, in the case of service-learning exercise programs for the disabled, in addition to programs based on sports activities, rehabilitation exercise and physical education programs to improve physical function are operated, so the results of other programs may differ from the results of this study. Third, although gender was not restricted, there is a possibility that there may be differences by gender because all of the recruited participants were boys.

Therefore, it would be appropriate to apply probability sampling to ensure the representativeness of the research participant group, and it would be necessary to include female children. Additionally, a comparison between a sports-based service-learning program and a rehabilitation exercise-based program for the disabled would be a more valuable study.

## 5. Conclusions

In conclusion, this study compared emotional and behavioral variables between a group of children with developmental disabilities who participated in a service-learning exercise program and a group that did not participate in service learning. This study was conducted to determine the impact of participation in a service-learning exercise program on the emotional and behavioral problems of children with developmental disabilities, and the following conclusions could be drawn: first, the service-learning exercise program had a positive effect on the internalization of problem behaviors and attention/hyperactivity scales in the emotional domain of children with developmental disabilities. Second, the service-learning exercise program positively affected the Personal Adjustment Scale in the problematic behavior area of children with developmental disabilities. Based on these results, participation in the service-learning exercise program may have a positive impact on the emotional and behavioral problems of children with developmental disabilities. Therefore, this could provide a basis for future research on service-learning exercise programs for children with developmental disabilities.

## Figures and Tables

**Table 1:** General characteristics of the participants.

Variables	SLG (n = 18)	Non-SLG (n = 18)	*p*
Age, years	11.5 ± 0.8	11.6 ± 0.9	0.841
Height, cm	151.8 ± 3.6	150.9 ± 3.4	0.526
Weight, kg	51.4 ± 4.1	50.9 ± 3.9	0.487

SLG, service-learning group.

**Table 2:** Program contents of service-learning exercise intervention.

Stage	Activity Content and Method
Preparation	Identify the characteristics of participants and evaluate their emotions and behavior Check basic physical functions and soccer skills
Stage 1 (1 week)	Soccer basic skill: Physical activity and interest-inducing activities to prevent injury (e.g., playing with a ball using hands and feet, wearing protective gear, rule explanation)
Stage 2 (2–3 weeks)	Soccer coordination: Physical activities for physical development (e.g., ball control, running, changing directions, and passing obstacles)
Stage 3 (4–5 weeks)	Technical training: Soccer skills training (e.g., ball dribbling, passing, shooting, offense and defense)
Stage 4 (6–12 weeks)	Game activities: Experience various sports situations through soccer games

**Table 3:** Effects of exercise on emotional problems.

Scale		Groups	Pre	Post	Group X Time
School problems	Attitude to school	SLG	47.53 ± 8.13	47.23 ± 8.79	F = 0.243 *p* = 0.765
		Non-SLG	48.12 ± 8.41	48.09 ± 9.12	
	Attitude to teacher	SLG	46.55 ± 7.31	45.11 ± 7.90	F = 0.381 *p* = 0.671
		Non-SLG	46.67 ± 6.88	46.88 ± 7.20	
	(Total) School problems	SLG	46.67 ± 7.76	45.53 ± 8.12	F = 0.287 *p* = 0.712
		Non-SLG	47.12 ± 7.43	47.57 ± 8.45	
Internalizing problems	Atypicality	SLG	48.60 ± 4.11	46.12 ± 4.55	F = 1.821 *p* = 0.311
		Non-SLG	48.98 ± 4.87	48.02 ± 4.29	
	Locus	SLG	48.32 ± 6.12	44.98 ± 6.12	F = 7.214 *p* = 0.002 *
		Non-SLG	48.10 ± 5.42	51.22 ± 9.38	
	Social stress	SLG	50.12 ± 5.89	46.89 ± 7.12	F = 3.911 *p* = 0.053
		Non-SLG	50.91 ± 6.08	49.41 ± 6.33	
	Anxiety	SLG	50.81 ± 4.87	45.12 ± 6.91	F = 8.289 *p* < 0.001 *
		Non-SLG	50.99 ± 5.12	51.19 ± 5.98	
	Depression	SLG	50.91 ± 5.27	46.11 ± 5.98	F = 7.191 *p* = 0.003 *
		Non-SLG	50.66 ± 6.12	51.71 ± 5.79	
	Sense of Inactivity	SLG	48.48 ± 6.12	46.41 ± 7.01	F = 4.181 *p* = 0.018 *
		Non-SLG	48.76 ± 5.81	50.31 ± 5.12	
	(Total) Internalizing problems	SLG	49.89 ± 5.89	46.12 ± 6.87	F = 4.291 *p* = 0.011 *
		Non-SLG	49.98 ± 5.76	50.52 ± 6.21	
Inattention/Hyperactivity	Inattention	SLG	49.41 ± 5.98	45.22 ± 5.19	F = 3.929 *p* = 0.031 *
		Non-SLG	49.82 ± 5.81	49.91 ± 6.12	
	Hyperactivity	SLG	49.12 ± 6.29	45.17 ± 5.82	F = 6.998 *p* = 0.007 *
		Non-SLG	49.81 ± 6.32	51.44 ± 7.12	
	(Total) Inattention/Hyperactivity	SLG	49.28 ± 6.11	45.20 ± 5.39	F = 4.761 *p* = 0.012 *
		Non-SLG	49.81 ± 6.02	50.49 ± 6.55	

* *p* < 0.05; SLG, service-learning group.

**Table 4:** Effects of the exercise intervention on behavioral problems.

Scale		Groups	Pre	Post	Group X Time
Personal adjustment	Relations with parents	SLG	50.38 ± 4.55	51.12 ± 4.98	F = 0.519 *p* = 0.691
		Non-SLG	50.12 ± 4.51	50.42 ± 4.61	
	Interpersonal relations	SLG	50.19 ± 5.45	54.89 ± 5.27	F = 6.841 *p* = 0.004 *
		Non-SLG	49.84 ± 5.31	49.13 ± 5.78	
	Self-esteem	SLG	50.09 ± 5.26	54.95 ± 6.19	F = 7.982 *p* = 0.002 *
		Non-SLG	50.72 ± 5.69	49.99 ± 5.53	
	Self-reliance	SLG	50.31 ± 5.28	53.89 ± 5.13	F = 6.921 *p* = 0.003 *
		Non-SLG	50.21 ± 5.41	48.29 ± 6.01	
	(Total) Personal adjustment	SLG	50.18 ± 5.07	53.89 ± 5.64	F = 4.116 *p* = 0.023 *
		Non-SLG	50.02 ± 5.12	49.35 ± 5.29	

* *p* < 0.05; SLG, service-learning group.

## Data Availability

The data presented in this study are available upon request from the corresponding author.
